# MicroRNA-495 downregulates AQP1 and facilitates proliferation and differentiation of osteoblasts in mice with tibial fracture through activation of p38 MAPK signaling pathway

**DOI:** 10.1038/s41598-019-50013-6

**Published:** 2019-11-07

**Authors:** Lei Zhu, Zun-Wen Lin, Gang Wang, Hong Zhang, Ben Liu, Qing-Jia Xu

**Affiliations:** 1 Department of Hand Surgery, Department of Foot and Ankle Surgery, Department of Orthopedics, Qilu Hospital of Shandong University, Jinan, 250012 P.R. China; 2 Department of Orthopedics, The First Affiliated Hospital of Nanchang University, Nanchang, 330006 P.R. China

**Keywords:** Cell biology, Diseases

## Abstract

Osteoblasts are implicated in the building of the vertebrate skeleton. The current study aimed to investigate the role of microRNA-495 (miR-495) in the osteoblasts of mice with tibial fractures and the underlying mechanism involving in aquaporin-1 (AQP1) and the p38 mitogen-activated protein kinase (p38 MAPK) signaling pathway. Initially, a microarray-based analysis was performed to screen the differentially expressed genes and miRNAs associated with tibial fracture. Following the establishment of a tibial fracture mouse model, the positive rate of the AQP1 protein in the fracture tissue was detected by immunohistochemistry (IHC). Next, to verify the binding site between miR-495 on AQP1, bioinformatics data were employed in addition to the application of a dual-luciferase reporter gene assay. The osteoblast cell line MC3T3-E1 was treated with miR-495 mimic, miR-495 inhibitor and Anisomycin to explore the potent effects of miR-495 on proliferation and differentiation of osteoblasts in mice with tibial fracture. The expression of miR-495, AQP1, p38 MAPK, PCNA, Cyclin D1, OCN, and OPN was subsequently evaluated by RT-qPCR and Western blot analysis. Cell viability, the number of calcium nodules and alkaline phosphatase (ALP) activity were detected by MTT assay, alizarin red staining, and ALP activity assay, respectively. Our results revealed that miR-495 was down-regulated while AQP1 was up-regulated in the mice with tibial fractures. AQP1 was verified as a target gene of miR-495. When the cells were treated with overexpressed miR-495 or activated p38 MAPK signaling pathway, elevated expression of PCNA, Cyclin, D1, OCN, and OPN along with an increased amount of calcium nodules, higher cell viability, and enhanced ALP activity was detected, while the expression of AQP1 was reduced. Collectively, the key findings of the present study support the notion that overexpressed miR-495 may activate the p38 MAPK signaling pathway to inhibit AQP1 and to promote the proliferation and differentiation of osteoblasts in mice with tibial fracture.

## Introduction

Tibia fracture is an injury often accompanied by long periods of immobility and disuse which may consequently lead to a permanent reduction in bone mineral density (BMD), predisposing an individual to osteoporotic fractures in later life^1^. However, the process of bone formation, remodeling and healing is a complex one. The coordinative role of various types of cell types has been linked to the process of bone formation, remodeling and healing^2^. The formation of bone is a highly regulated and intricate developmental process, which relates to the differentiation of mesenchymal stem cells into osteoblasts^3^. Osteocytes, which consist of more than 90% of the cells in bone, are derived from osteoblasts^4^. More recently, mice have been increasingly employed to investigate the mechano-biology of fracture healing^5^.

Based on the information from the GSE494 chip, aquaporin-1 (AQP1) was found to be highly expressed in impaired fracture healing when compared with that of normal fracture healing. AQP1, one of the AQP water channels which are critical to water homeostasis in all organisms, is specifically localized to Schwann cells in the peripheral nervous system (PNS)^6,7^. Besides, we conducted bioinformatics prediction in order to select the miRNA capable of regulating AQP1. MiRNAs, single-stranded non-coding RNAs, could down-regulate the expression of target genes through the degradation or translational inhibition of either mRNA, which exists in many organisms^8^. Accumulating studies have highlighted the role of microRNAs (miRNAs) in osteoblast differentiation and bone formation, such as miR-2861 and miR-142-3p^9,10^. Furthermore, miR-495 has been revealed to play a notable role in the proliferation and differentiation of osteoblasts^11^. The signal transduction of p38 mitogen-activated protein kinase (p38 MAPK) signaling pathway, a common pathway of intracellular signaling transduction after extracellular, has a strong correlation with the growth, development, proliferation, and apoptosis of cells^12^. A study has indicated the role of the p38 MAPK signaling pathway in the down-regulation of AQP1^13^. Furthermore, it has been reported that the activation of p38 MAPK signaling pathway possesses the ability to facilitate the expression of osteogenic gene^14^. Interestingly, the key role of osteocytes in bone remodeling has been detected in osteocytes differentiated from osteoblasts^15^. Hence, we hypothesized that miR-495 may have effects on the osteoblast proliferation and differentiation through the p38 MAPK signaling pathway. Hence, during the current study we aimed to investigate the capacity of miR-495 to promote the proliferation and differentiation of osteoblasts in mice with tibial fracture through the p38 MAPK signaling pathway.

## Results

### MiR-495 affects fracture healing via regulating AQP1

**Figure 1:**
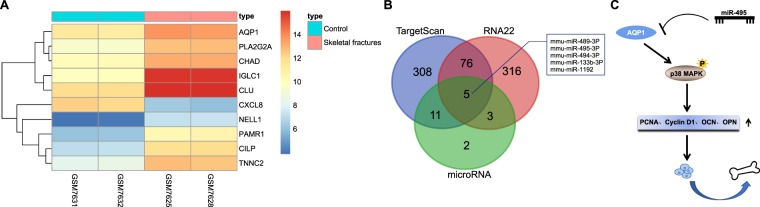
MiR-495 affects fracture healing via regulating AQP1. (**A**) The heat map of the top 10 differentially expressed genes of GSE494 gene expression dataset; (**B**) MicroRNA, RNA22 and TargetScan predicted mmu-miR-489-3p, mmu-miR-495-3p, mmu-miR-494-3p, mmu-miR-133b-3p and mmu-miR-1192 may have targeting effects on AQP1; (**C**) the mechanism image of miR-495 affecting differentiation of osteoblasts via regulation on AQP1. miR-495, microRNA-495; AQP1, aquaporin-1.

In accordance with the data of the GSE494 chip, a total of 185 differentially expressed genes related to fracture healing were screened out and the top 10 was identified using a heat map (Fig. 1A). AQP1 exhibited a higher expression in impaired fracture healing when compared to normal fracture healing. Previous data has suggested that AQP1 plays a contributory role in the progression of osteosarcoma, multiple myeloma and rheumatoid arthritis through its effects on bone marrow mesenchymal stem cells and synovial tissue^16–18^. The role of AQP1 in fracture healing remains unclear. The number of miRNA targeting AQP1 was 21 predicted by MicroRNA, 750 by RNA22 and 504 by TargetScan. In TargetScan, context ++ score is an indicator to evaluate predicted target efficacy^19^. Based on the ascending order of context ++ score, the top 400 predicted miRNAs were obtained from TargetScan. Besides, the top 400 predicted miRNAs of RNA22 were acquired based on default sort. The predicted miRNAs were intersected, the results of which revealed that there were five intersecting miRNAs, including mmu-miR-489-3p, mmu-miR-495-3p, mmu-miR-494-3p, mmu-miR-133b-3p and mmu-miR-1192 (Fig. 1B). A lower mirSVR score reflected stronger miRNA-mRNA combination. In this case, a corresponding miRNA was more likely to down-regulate genes, which was considered as a threshold for miRNA-mRNA prediction^20^. In the microRNA database, mmu-miR-495 was identified to have the lowest mirSVR score. The results indicated that miR-495 could potentially influence fracture healing via its regulation on AQP1 and then can be regarded as a promising target for fracture treatment (Fig. 1C).

### Histopathological observation of osteogenic tissue in mice with fracture

**Figure 2:**
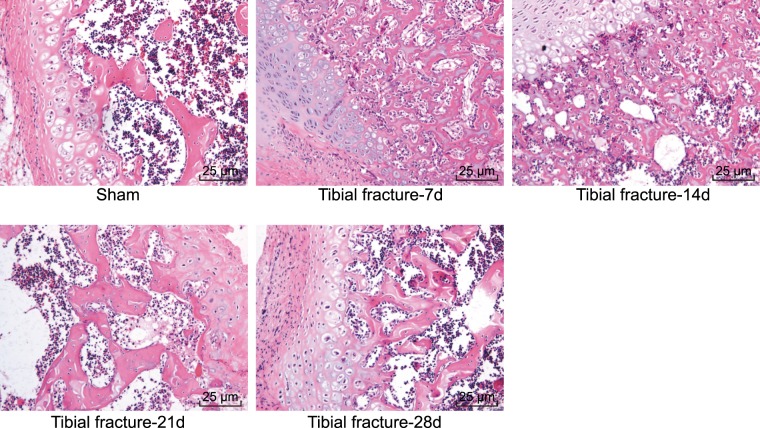
Pathological observation of tissues in the five groups (×400), and twenty-eighth days after fractured in mice, the callus was reshaped, gradually formed the medullary cavity and healed completely. HE, hematoxylin-eosin.

HE staining was applied to observe the histopathological features of the tissues from the five groups. A distinct medullary cavity with closely arranged cells situated around the medullary cavity was identified in the sham group. Results of fracture group were as follow: seven days after fractures were established in the mice, periosteal thickening and a hard callus were detected on the broken ends of the lateral cortex bone fracture area; both sides of the fracture zone were filled with a significant amount of undifferentiated mesenchymal cells, with the mesenchymal cells found to be differentiating into chondrocytes, with signs of hypertrophy identified in the endochondral bone region of the callus. Fourteen days after the fractures had been established in mice, the greater majority of the chondrocytes had been absorbed with signs of calcification detected in the cartilage. Twenty-one days after fractures had been established, the bone callus region was found to be filled with woven bone while the intramembranous osteogenesis area exhibited osseous callus. Twenty eight days after the fractures had been established in the mice, and the callus was reshaped, gradually formed the medullary cavity and healed completely (Fig. 2).

### The positive expression of AQP1 is higher in mice with fracture

**Figure 3:**
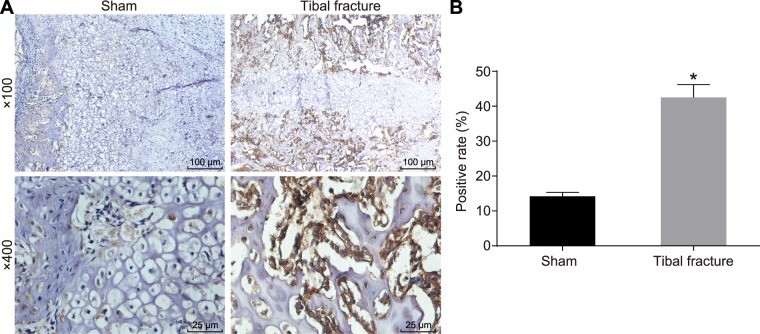
Positive expression of AQP1 is higher in mice with fracture. (**A**) AQP1 expression in the sham group and the tibial fracture group detected using IHC (400x); (**B**) the positive expression rate of AQP1 protein in the sham group and the tibial fracture group; *p < 0.05 *vs*. the sham group; AQP1, aquaporin-1.

The next step was to determine the positive expression of AQP1 in the mice with fractures using IHC, the results of which are displayed in Fig. 3. The positive expression of AQP1 was reflected by dark yellow or brown in fracture tissues of mice on the 7^th^ day, mostly expressed in the cell membrane as well as a small amount in the cytoplasm. The positive expression rate of AQP1 protein in the fracture tissues of mice was 42.5 ± 3.7%, which was significantly higher than that in the sham group (14.2 ± 1.1%) (*p* < 0.05). The aforementioned results suggest that the positive expression of AQP1 may be up-regulated in mice with fracture.

### Mouse fracture model is successfully established

Next, the maximum load, elastic modulus and elastic deflection were detected. As depicted in Table 1, compared with the sham group, on the 7^th^, 14^th^ and 21^st^ days after fracture, the maximum load, elastic modulus and elastic deflection of tibia were significantly decreased (*p* < 0.05) and the maximum load, elastic modulus and elastic deflection exhibited a gradual upward inclination with the increase of time; on the 28^th^ day after fracture; there was no significant difference identified between the sham group and the model group in terms of the maximum load, elastic modulus and elastic deflection (*p* > 0.05).

**Table 1:** Structural mechanics performance indexes of tibia in each group.

Group	Maximum load (N)	Elastic modulus (GPa)	Elastic deflection (mm)
sham	8.55 ± 0.41	6.41 ± 0.54	0.67 ± 0.05
Model (Day 7)	3.75 ± 0.40^*^	3.32 ± 0.27^*^	0.40 ± 0.03^*^
Model (Day 14)	5.69 ± 0.45^*^	4.75 ± 0.33^*^	0.53 ± 0.04^*^
Model (Day 21)	7.22 ± 0.53^*^	5.61 ± 0.39^*^	0.61 ± 0.04^*^
Model (Day 28)	8.32 ± 0.44	6.28 ± 0.51	0.64 ± 0.05

Note: *p < 0.05 *vs*. the sham group.

### AQP1 expression is up-regulated and the expression of miR-495, p38 MAPK, PCNA, Cyclin D1, OCN and OPN down-regulated in mice with fracture

**Figure 4:**
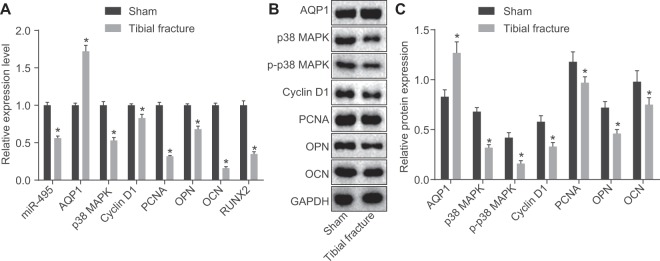
AQP1 expression is elevated yet miR-495, p38 MAPK, PCNA, Cyclin D1, OCN and OPN expression reduced in mice with tibial fracture. (**A**) Relative miR-495 expression and mRNA expression of AQP1, p38 MAPK, PCNA, Cyclin D1, OCN and OPN in the sham group and the tibial fracture group; (**B**) grey value analysis of relative genes in the sham group and the tibial fracture group; (**C**) relative protein levels in the sham group and the tibial fracture group; *p < 0.05 *vs*. the sham group; miR-495, microRNA-495; AQP1, aquaporin-1; p38 MAPK, p38 mitogen-activated protein kinase; PCNA, proliferating cell nuclear antigen; OCN, osteocalcin; OPN, steopontin; p-p38 MAPK, phosphorylation p38 mitogen-activated protein kinase.

RT-qPCR and Western blot analysis were employed to determine the expression of miR-495, AQP1, p38 MAPK, p-p38 MAPK, PCNA, Cyclin D1, OCN and OPN in mice with fracture (Fig. 4A–C). Compared with the sham group, the expression of miR-495 as well as the mRNA and protein levels of p38 MAPK, PCNA, Cyclin D1, OCN, and OPN in the tibial fracture group was markedly decreased, while AQP1 mRNA and the protein levels significantly increased (all *p* < 0.05). The above data indicated that AQP1 may be highly expressed while miR-495, p38 MAPK, PCNA, Cyclin D1, OCN, and OPN lowly expressed in mice with tibial fracture.

### AQP1 is a target gene of miR-495

**Figure 5:**
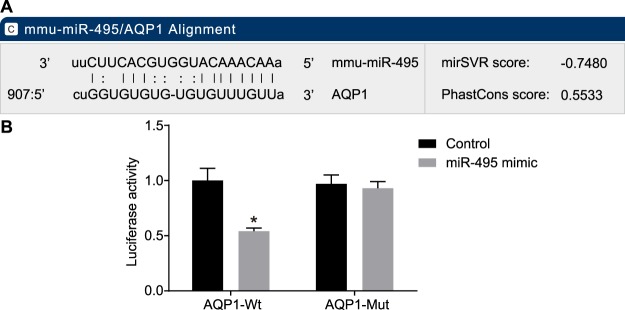
Biological prediction website and dual-luciferase reporter gene assay verify miR-495 targets and negatively regulates AQP1. (**A**) predicted binding site of miR-495 in AQP1-3′UTR; (**B**) luciferase activity of cells transfected with AQP1-3′UTR-Wt and AQP1-3′UTR-Mut; *p < 0.05 *vs*. the control group; miR-495, microRNA-495; AQP1, aquaporin-1; UTR, untranslated region; Wt, wild type; Mut, mutant type.

In order to explore the relationship between miR-495 and AQP1, we used biological prediction website and dual-luciferase reporter gene assay. The biological prediction website available at http://www.microrna.org suggested that AQP1 was a target gene of miR-495 (Fig. 5A). The results of the dual-luciferase reporter gene assay demonstrated that the luciferase activity was significantly descended in the miR-495 and AQP1-Wt co-transfection group compared with the control group (*p* < 0.05). However, in cells carried with the AQP1-Mut plasmid, luciferase activity between the two groups exhibited no significant difference (*p* > 0.05). These findings provided evidence suggesting that AQP1 is a direct target gene of miR-495 (Fig. 5B).

### MiR-495 overexpression or p38 MAPK signaling pathway activation promotes osteoblast proliferation and differentiation

**Figure 6:**
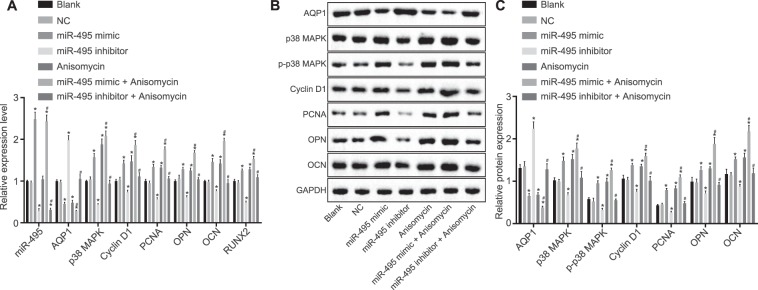
miR-495 overexpression or p38 MAPK signaling pathway activation contributes to osteoblast proliferation and differentiation and inhibited AQP1 in mice with tibial fracture. (**A**) Relative miR-495 expression and mRNA expression of AQP1, p38 MAPK, PCNA, Cyclin D1, OCN and OPN in MC3T3-E1 cells after transfection; (**B**) grey value analysis of AQP1, p38 MAPK, p-p38 MAPK, PCNA, Cyclin D1, OCN and OPN in MC3T3-E1 cells after transfection; (**C**) protein levels of AQP1, p38 MAPK, p-p38 MAPK, PCNA, Cyclin D1, OCN and OPN in MC3T3-E1 cells after transfection; *p < 0.05 *vs*. the blank group; ^#^*p* < 0.05 *vs*. the miR-495 mimic and Anisomycin groups; miR-495, microRNA-495; AQP1, aquaporin-1; p38 MAPK; p38 mitogen-activated protein kinase; PCNA, proliferating cell nuclear antigen; OCN, osteocalcin; OPN, osteopontin; RUNX2, runt-related transcription factor 2.

To investigate the involvement of miR-495 and p38 MAPK signaling pathway in the osteoblast proliferation, RT-qPCR was applied to detect the miR-495 expression and mRNA expression of AQP1, p38 MAPK, PCNA, RUNX2, Cyclin D1, OCN, and OPN in osteoblast cell line MC3T3-E1 after 48 h of transfection. As illustrated in Fig. 6A, no significant difference was detected regarding the expression of miR-495 as well as the mRNA expression of AQP1, p38 MAPK, PCNA, RUNX2, Cyclin D1, OCN, and OPN in the blank and NC groups (all *p* > 0.05). Compared with the blank group, miR-495 expression and the mRNA expression of p38 MAPK, PCNA, RUNX2, Cyclin D1, OCN, and OPN were markedly elevated in the miR-495 mimic group, while the mRNA expression of AQP1 was notably decreased (all *p* < 0.05); while the miR-495 inhibitor group displayed an opposite trend. The expression of miR-495 exhibited no changes, while the mRNA expression of p38 MAPK, PCNA, RUNX2, Cyclin D1, OCN, and OPN displayed considerable increases along with a reduced mRNA expression of AQP1 in the Anisomycin group when compared to the blank group. In the miR-495 mimic + Anisomycin group, upregulated miR-495 expression as well as increased mRNA expression of p38 MAPK, PCNA, RUNX2, Cyclin D1, OCN, and OPN was detected, while AQP1 was downregulated in comparison with the blank group (all *p* < 0.05). The miR-495 inhibitor + Anisomycin group exhibited downregulated miR-495 (*p* < 0.05) and unchanged mRNA expression of AQP1, p38 MAPK, PCNA, RUNX2, Cyclin D1, OCN, and OPN (all *p* > 0.05) in contrast to the blank group.

Next, Western blot analysis was used to detect the protein levels of AQP1, p38 MAPK, PCNA, RUNX2, Cyclin D1, OCN, and OPN. As displayed in Fig. 6B-C, there was no significant difference in the protein levels of AQP1, p38 MAPK, PCNA, RUNX2, Cyclin D1, OCN, and OPN between the blank, NC and miR-495 inhibitor + Anisomycin groups (all *p* > 0.05). Compared with the blank group, the protein levels of p38 MAPK, PCNA, RUNX2, Cyclin D1, OCN, and OPN as well as the extent of p-p38 MAPK were all notably elevated, while the AQP1 protein level was notably decreased in the miR-495 mimic, Anisomycin and miR-495 mimic + Anisomycin groups (the miR-495 mimic + Anisomycin group exhibited a more significant upward trend than that of the miR-495 mimic and Anisomycin groups) (all *p* < 0.05); while the miR-495 inhibitor group exhibited an opposite trend. The aforementioned findings demonstrate that miR-495 overexpression or p38 MAPK signaling pathway activation can inhibit the expression of AQP1 and promote osteoblast proliferation and differentiation.

### MiR-495 overexpression or p38 MAPK signaling pathway activation promotes MC3T3-E1 cell proliferation

**Figure 7:**
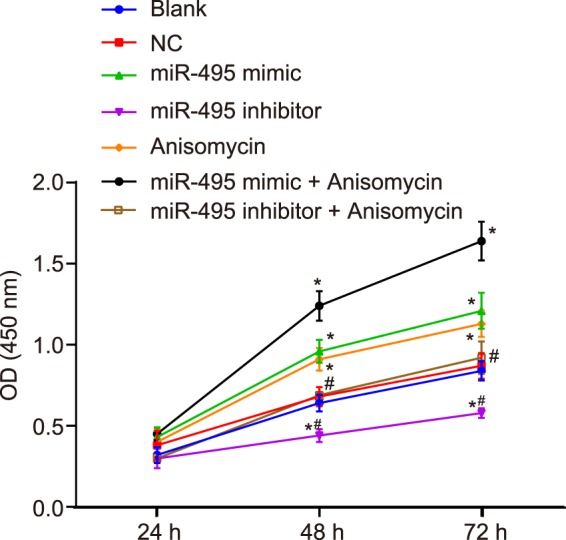
miR-495 overexpression or p38 MAPK signaling pathway activation can inhibit MC3T3-E1 cell proliferation. *p < 0.05 *vs*. the blank group; ^#^*p* < 0.05 *vs*. the miR-495 mimic and Anisomycin groups; miR-495, microRNA-495; AQP1, aquaporin-1; NC, negative control; OD, optical density.

Next, MTT assay was employed to investigate the role of miR-495 and p38 MAPK signaling pathway in cell viability of MC3T3-E1 cells after 48 h of transfection. The results obtained indicated that (Fig. 7), there was no significant difference in cell proliferation at 24 h in each group. Additionally, there was no significant difference in cell viability between the NC, blank and miR-495 inhibitor + Anisomycin groups at different time points (*p* > 0.05). Compared with the blank group, the cell viability in the miR-495 mimic and Anisomycin groups was notably increased at 48 h and 72 h (*p* < 0.05), while notably decreased in the miR-495 inhibitor group (all *p* < 0.05). The ascending cell viability trend in the miR-495 mimic + Anisomycin group was more distinct than that in the miR-495 mimic and Anisomycin groups. Taken together, the results obtained suggest that the over-expression of miR-495 or activation of the p38 MAPK signaling pathway can promote MC3T3-E1 cell proliferation.

### MiR-495 overexpression or p38 MAPK signaling pathway activation promotes the formation of calcium nodule of MC3T3-E1 cells

**Figure 8:**
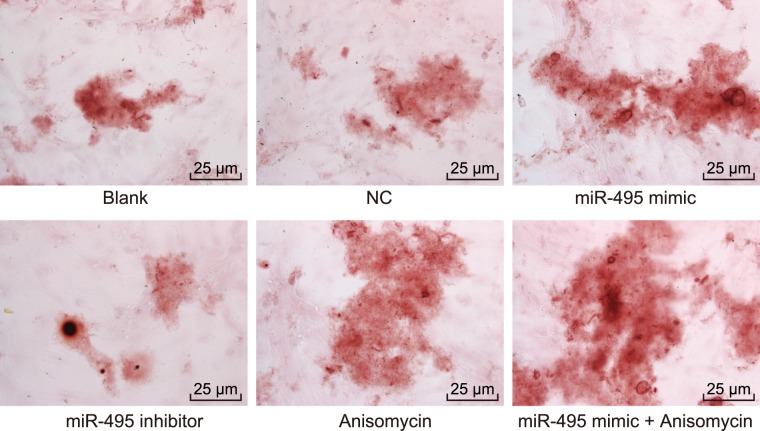
miR-495 overexpression or p38 MAPK signaling pathway activation accelerates the formation of calcium nodule of osteoblast MC3T3-E1 in fracture mice (400×). miR-495, microRNA-495; AQP1, aquaporin-1; NC, negative control.

In order to explore the effects associated with the overexpression of miR-495 or p38 MAPK signaling pathway activation on osteoblast differentiation, alizarin red staining was applied to measure the formation of calcium nodule of MC3T3-E1 cells after 48 h of transfection. The results of the alizarin red staining (Fig. 8) under the microscope revealed red calcium nodules on the 21^st^ day. No significant difference between the NC, blank and miR-495 inhibitor + Anisomycin groups was detected regarding the amount of calcium nodules (*p* > 0.05). Compared with the blank group, the amount of calcium nodules was markedly elevated in the miR-495 mimic, Anisomycin and miR-495 mimic + Anisomycin groups (*p* < 0.05) The ascending trend in the miR-495 mimic + Anisomycin was more distinct than that in the miR-495 mimic and Anisomycin groups. The amount of calcium nodules in the miR-495 inhibitor group was notably lower than that in the blank group (*p* < 0.05). All these results indicate that the over-expression of miR-495 or activation of the p38 MAPK signaling pathway accelerates the formation of calcium nodule of osteoblast MC3T3-E1 in fracture mice.

### MiR-495 overexpression or p38 MAPK signaling pathway activation facilitates ALP activity

**Figure 9:**
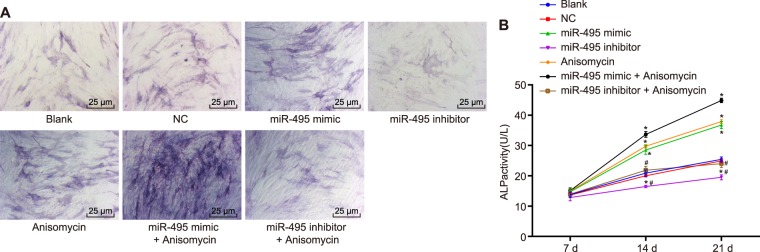
ALP activity assay manifests miR-495 overexpression or p38 MAPK signaling pathway activation facilitates ALP activity. (**A**) ALP staining image in each group (400×); (**B**) ALP activity in each group; *p < 0.05 *vs*. the blank group; ^#^*p* < 0.05 *vs*. the miR-495 mimic and Anisomycin groups; miR-495, microRNA-495; AQP1, aquaporin-1, NC, negative control.

Finally, in a bid to detect the effects of miR-495 and p38 MAPK signaling pathway on ALP activity, we performed ALP activity assay on MC3T3-E1 cells after 48 h of transfection. The results obtained indicated that ALP activity was elevated as time increased among the cells in each group. The blank, NC and miR-495 inhibitor + Anisomycin groups exhibited no remarkable difference in the ALP activity at each time point (*p* > 0.05). From the 14^th^ day to the 21^st^ day, compared with the blank group, the ALP activity increased significantly in the miR-495 mimic, Anisomycin and miR-495 mimic + Anisomycin groups (*p* < 0.05), while the ascending trend in the miR-495 mimic + Anisomycin group was more significant than that in the miR-495 mimic and Anisomycin groups; the ALP activity of cells in the miR-495 inhibitor group decreased significantly (*p* < 0.05) (Fig. 9). Taken together, the aforementioned data suggest that overexpressed miR-495 or activated p38 MAPK signaling pathway can enhance the ALP activity of osteoblast MC3T3-E1 in mice with tibial fracture.

## Discussion

Fracture healing, is a process clearly associated with osteoblast differentiation. Altered osteoblast differentiation has been highlighted in patients who develop recurrent and recalcitrant fracture non-unions^21^. MiR-495, as a promoter of osteoblast proliferation and differentiation has been largely investigated recently. Xu X *et al*. reported that miR-495 promoted the proliferation and differentiation of the osteoblasts^11^. Besides, Tian, Z.*et al*. found that the osteogenesis and osteoblast apoptosis were correlated with miR-495, whose target gene was HMGA2 in this process^22^. The current study aimed to elucidate the effects of miR-495 on osteoblast proliferation and differentiation in mice with tibial fracture and its effects on AQP1 gene as well as the p38 MAPK signaling pathway. Key findings of this study demonstrated that miR-495 activates the p38 MAPK signaling pathway, thus inhibiting AQP1 and promoting proliferation and differentiation of osteoblasts in mice with tibial fracture.

The obtained findings revealed that miR-495 played a crucial role in the proliferation and differentiation of the osteoblasts in mice with tibial fracture. MiR-495 was observed to be overexpressed during mesenchymal stem cell (MSC) differentiation into osteoblasts while a reduction in its expression has been identified during the process of differentiation into adipocytes^11^. Janet D. *et al*. proposed that miR-495 likely functions as a tumor suppressor in acute myeloid leukemia (AML) with mixed lineage leukemia (MLL) rearrangements by targeting essential leukemia-related genes^23^. More specifically, miR-495 has been reported to exert notable effects on the proliferation and differentiation of osteoblasts predominately through its inhibitory effects on the targeted genes, such as glucose-regulated protein 78 (Grp78), growth factor independent 1 (GFi-1) and runt-related transcription factor 3 (RUNX3)^24–27^. Another key observation of the current study indicated that miR-495 targeted AQP1. Interestingly, the postulation that AQP1 is a putative target gene of miR-495 was further validated by the results obtained from the online analysis software http://www.microRNA.org. Recently, the aquaporin family was found to be related to osteoblasts and bone fracture. For instance, the expression of AQP5 is found in the bone marrow-derived MSCs (BMMSCs) and AQP5 could facilitate its apoptosis^28^. Hypoxia-inducible factor-1α (HIF-1α) positively regulates AQP4, as reported by Higashida T *et al*. highlighting the negative effect of HIF-1α on the progression of osteoblasts^29,30^. Hence, we suspected that AQP1 is associated with osteoblasts. In the following experiments, our results illustrated that the function of miR-495 influencing the proliferation and differentiation of osteoblasts in mice with tibial fractures was achieved by targeting AQP1 *via* the p38 MAPK signaling pathway. The p38 MAPK signaling pathway was observed to play a major role in the regulation of the development skeletal muscle^31^. A correlation between AQP1 and the p38 MAPK signaling pathway has been suggested. Yang M *et al*. suggested that the MAPK signaling pathway was responsible for the regulation of aquaporins (AQPs), including AQP1^32^. Furthermore, Liu L and Xie C emphasized the importance of p38 MAPK in downregulating the expression of AQP1 via peptidoglycan (PGN) in MeT-5A cells^33^. Meanwhile, the down mediation of AQP1 could be truly achieved by the p38 MAPK signaling pathway^34^. To conclude, the p38 MAPK signaling pathway plays a critical role in the down regulation of AQP1. Notably, activation of p38 MAPK signaling pathway has been reported to enhance the proliferation and differentiation of osteoblasts by promoting the expression of osteogenic gene^14^. Moreover, berberine (BBR) has been shown to alleviate osteoporosis, and stimulate bone area formation in calvarial organ culture through its role in stimulating osteoblast differentiation by activating RUNX2 by p38 MAPK^35^. Activation of the p38 MAPK signaling pathway was found to enhance osteoblast proliferation and differentiation in fracture mice.

## Conclusion

Taken together, the key findings of the study revealed that miR-495 is poorly expressed and acts as a promoter in the proliferation and differentiation of osteoblasts and an inhibitor of AQP1 in mice with tibial fracture. The action of miR-495 depends on activation of the p38 MAPK signaling pathway. Based on our observations, we believe that miR-495 could be a promising therapeutic target for fracture treatment. Although other complex factors and signaling pathways are involved in the regulation of osteoblast metabolism, our findings may still help to elucidate the specific mechanism by which miR-495 exerts a protective effect against fractures by binding to AQP1 *via* the p38 MAPK signaling pathway.

## Materials and Methods

### Ethics statement

All animal experiments were conducted with the approval of Animal Ethics Committee of Qilu Hospital of Shandong University. All experiments were performed in strict accordance with relevant guidelines and regulations. Extensive efforts were made to minimize animal usage and suffering.

### Microarray-based analysis

Through Gene Expression Omnibus (GEO) database (http://www.ncbi.nlm.nih.gov/geo/), gene expression dataset related to tibial fracture was retrieved using “skeletal fracture” as the key words. Fracture gene expression dataset (GSE494) and annotate probe files were downloaded, which were obtained from GPL8300 [HG_U95Av2] Affymetrix Human Genome U95 Version 2 Array. GSE494 contains gene expression of fracture patient with or without normal fracture healing. Next, differentially expressed genes influencing fracture healing were screened with normal fracture healing as the control group. The Limma package of R language was applied in order to conduct the standard pretreatment and screen the differentially expressed genes with *P*.*Value* < 0.05 and |LogFoldChange| >2 set as the threshold^36^, and a thermal map of differentially expressed genes was drawn. MicroRNA (http://34.236.212.39/microrna/getGeneForm.do), RNA22 tool (https://cm.jefferson.edu/rna22/) and TargetScan (http://www.targetscan.org/vert_71/) were regarded as widely used miRNA-gene interaction prediction software, which could predict not only the target relationship but also the binding sites between miRNAs and genes. As a result, they were employed to screen out the target miRNA for differentially expressed genes.

### Model establishment

Specific-pathogen-free (SPF) C67BL/6 mice (weight: 22–28 g; aged: 8 weeks old; regardless of gender) were purchased from the Experimental Animal Center of the Xuanwu hospital, Capital Medical University (Beijing, China). The mice were housed in cages (5 per cage) and granted free access to food and water. The cage environment was controlled with the temperature set at 22–25 °C with a humidity of 50–60% as well as a 12 h/12 h light-dark illumination schedule. After one week’s adaptation, the tibial fracture mouse model was established. One day prior to the operation, the operation room was disinfected with ultraviolet rays overnight. The procedures of the model establishment were performed according to the following: after intraperitoneal anesthesia with pentobarbital sodium (2%, 60 mg/kg) in mice, the hair located on the mice’s left leg was shaved and the left leg was disinfected using alcohol. The sterile surgical towels were spread, after which an incision was made in the anterior region of the left tibia to expose the tibial shaft (about 1 cm in length) and an electric drill was performed above the tibial tubercle. Subsequently, the Kirschner wire (1 mm) was inserted into the medullary cavity direction. Next, the surrounding soft tissues were dissected from the middle part of the tibia, with the tibia separated using a transverse saw. Finally, following reduction of the fracture, a Kirschner wire was inserted into the distal end, firmly fixed, and bent at the terminal end of the needle with the excess part cutting off; the incision was closed layer by layer. After the operation, 80 thousand units of penicillin sodium were injected intraperitoneally into each mouse for three days. Forty tibial fracture mice (mouse model with tibial fracture) were divided into 4 groups. On the 7^th^ day, 14^th^ day, 21^st^ day, 28^th^ day, respectively after operation, 10 mice in each group were collected. Five of them were used for making paraffin section of callus, while five for callus tissue samples and the other 10 were regarded as the sham group. After intraperitoneal anesthesia was successfully conducted with the injection of 2% pentobarbital sodium (60 mg/kg), the left leg of mice was shaved and sterilized with alcohol. The sterile surgical towels were spread, and the tibia of the left leg was excised right ahead to expose tibia shaft (about 1 cm). No further operation was conducted, and the wound was sutured.

### Hematoxylin-eosin (HE) staining

The callus tissues were collected on the 7^th^ day, fixed in 10% neutral formaldehyde for 24 h, dehydrated by alcohol, cleared by xylene, mounted by neutral balsam and imbedded in paraffin. Five-micrometer-thick tissue sections were collected from the formalin-fixed paraffin-embedded (FFPE) blocks. After dewaxing with xylene (twice, 10 min each time), the sections were embedded in absolute ethanol (5 min each), ethanol (90%, 2 min), ethanol (70%, 2 min), and washed with distilled water for 2 min. The sections were then stained with hematoxylin for 7 min, washed under running water (10 min), washed by distilled water, embedded in ethanol (95%, 5 s), and stained with eosin for 1 min. Absolute ethanol, 95% ethanol, 75% ethanol, and 50% ethanol (twice, 2 min each) were used for hydration, xylene (twice, 5 min each) for clearing, and neutral balsam for mounting. The histological changes were analyzed under an optical microscope (Nikon, Tokyo, Japan) and imaged.

### Immunohistochemistry (IHC)

The callus tissues in each group were collected on the 7^th^ day, fixed by 10% formalin and treated with xylene (twice, 10 min each time). Absolute ethanol, 95% ethanol, 75% ethanol, and 50% ethanol (each for 5 min) were used for hydration while distilled water (5 min) was used for washing. Next, 0.2 mol/L phosphate buffered saline (PBS) was added (5 min) for washing, and a drop of H_2_O_2_ was added for inactivation. The sections were incubated at room temperature for 10 min. The sections were subsequently washed with PBS for 9 min, soaked in 0.01 mol/L citrate buffer (PH 6.0) in microwave for 20 min for repair and rewashed by PBS for 9 min. One drop of normal goat serum was added to the sections which had been incubated at room temperature for 5 min. After the serum was removed, the rabbit anti-mouse AQP1 monoclonal antibody (1: 500, ab15080, Abcam, Cambridge, MA, USA) was added to the sections for overnight incubation at 4 °C and at 37 °C for 30 min, and then washed three times PBS (3 min per wash). The sections were then incubated with biotinylated goat anti-rabbit immunoglobulin G (IgG) secondary antibody (1: 1000, ab6789, Abcam, Cambridge, MA, USA) at 37 °C for 30 min. Next, the sections were washed 3 times with PBS (3 min per wash), stained with diaminobenzidine (DAB) chromogen (Boster Bioengineering Co., Ltd, Wuhan, China) for 1–2 min and rewashed three additional times with PBS (2 min per wash). Next, the sections were counterstained with hematoxylin (Nanjing KeyGen Biotech Co., Ltd., Nanjing, Jiangsu, China) for 1 min, dehydrated, cleared, mounted by balsam and analyzed under a microscope. The samples confirmed to be positive were regarded as the positive control, and the PBS instead of the primary antibody used as the negative control. Finally, 5 fields were randomly selected from each section for observation under a 100-fold upright optical microscope (Nikon, Tokyo, Japan), with 100 cells counted in each field. The positive cells were regarded as the cells with a staining degree greater than 25%, which was reflected by distinctive brown or yellow granules. Positive rate = (the number of positive cells/the number of total cells) × 100%.

### Biomechanical examination

On the 7^th^ day, 14^th^ day, 21^st^ day and 28^th^ day after operation, an additional 5 mice from each group were euthanized by cervical dislocation, followed by femur collection, with the muscle and fascia removed. The intramedullary pins were then cautiously removed. After microcomputed tomography (Micro CT) scanning, the femur specimens were wrapped in normal saline gauze, placed in sealed sample bag and stored in a freezer at 20 °C. The femur specimens were removed 1 day prior to the experiment and thawed naturally at room temperature of 23 °C. The tibial bone was placed on the two scaffolds, with the distance between the two brackets set at 6 mm. The load was set at a rate of 10 mm/min loading speed, with the three point bending test performed at room temperature. The samples were kept moist during the experiment. A computer was used to draw the load deformation curve automatically and output the maximum load, elastic modulus and elastic deflection.

### Cell treatment

The osteoblast cell line MC3T3-E1 were grouped into a blank group (cells without any treatment), a negative control group (NC; cells treated with empty plasmid), a miR-495 mimic group (cells treated with 4 μg miR-495 mimic), a miR-495 inhibitor group (cells treated with 4 μg miR-495 inhibitor), a Anisomycin group (A9789-25mg; cells treated with 1 μM Anisomycin, activator of MAPK signaling pathway; Sigma-Aldrich, St. Louis, MO, USA), a miR-495 mimic + Anisomycin group (cells treated with 1 μM Anisomycin and 4 μg miR-495 mimic) and a miR-495 inhibitor + Anisomycin group (cells treated with 4 μg miR-495 inhibitor and 1 μM Anisomycin). All the target plasmids were purchased from Dharmacon (Lafayette, CO, USA). MC3T3-E1 cells were seeded in a 6-well plate at a density of 3 × 10^5^ cell/well. When cell confluence had reached approximately 80%, the Lipofectamine 2000 kit (Invitrogen, Carlsbad, CA, USA) was employed for transfection. Four μg target plasmids and 10 μL Lipofectamine 2000 were diluted in 250 µL serum-free Opti-MEM (Gibco BRL, Grand Island, NY, USA) at room temperature for 5 min and then fully mixed. Next, the mixture was permitted to stand for 20 min. After incubation, the mixture was seeded into the plates and cultured in a 5% CO_2_ incubator under saturated humidity conditions at 37 °C for 6 h. Finally, the cells were transferred to a complete medium for 48-h period of incubation, after which they were collected and stored for subsequent experiments.

### Dual-luciferase reporter gene assay

Bioinformatics software (http://www.microRNA.org) was employed to analyze the target gene of miR-495 and to determine whether AQP1 was a direct target of miR-495. The 3′untranslated region (3′UTR) of AQP1 gene (Accession Number: NM_007472.2; the sequence of the oligos used for this amplification is: Forward: 5′-ATGCGCTAGC GCCACGGATCAGAGAATCAG-3′; Reverse: 5′-GTGCTCTAGA TCAGACATGACACTGCACATAG-3′) was cloned, and the PCR products were cloned into the downstream of pmirGLO (Promega, Madison, WI, USA) luciferase vector using the endonuclease sites NheI and XbaI to build the AQP1-wild type (Wt) vector. Target gene database was used to predict the binding sites of miR-495 and its target genes, and site-specific mutagenesis was used to construct AQP1-mutant (Mut) vector (5′-CUGGU***AACGA***UG***ACCCAGAA***A-3′). Renilla luciferase expression vector pRL-TK (TaKaRa, Dalian, China) was regarded as the internal reference, after which the miR-495 mimic and NC were transfected into the cells with the luciferase reporter vectors respectively. The dual-luciferase reporter gene assay system (Promega, Madison, WI, USA) was employed to detect the luciferase activity.

### Reverse transcription-quantitative polymerase chain reaction (RT-qPCR)

Total RNA (about 100 mg) was extracted from tissues using the Trizol^TM^ Kit (No. 16096020, Thermo Fisher Scientific, New York, USA). The primers were designed with Primer Premier 5 and Oligo 6 biological software and synthesized by Shanghai Sangon Biotechnology Co. Ltd. (Shanghai, China) (Table 2). A total of 10 μL RNA samples were diluted a total of 20 times by ultrapure water without RNA enzyme, and the optical density (OD) values at 260 nm and 280 nm at ultraviolet spectrophotometer were used to evaluate the RNA concentration and purity. The amount of Total RNA was V = 1.25/OD260 (μL). Next, to detect the expression of miR-495, a total of 20 μL reverse transcription system was reversely transcribed as per the instructions of the TaqMan™ Advanced miRNA cDNA Synthesis Kit (A28007, Applied Biosystems, Foster City, CA, USA) Five μL Mix reagent (No. 4368702, Beijing Tideradar Technology Co., Ltd., Beijing, China), 5 μL total RNA and 10 μL RNase Free H_2_O were added into an Eppendorf (EP) tube and reacted in a PCR instrument after centrifugation and mixing. The reaction conditions were set as follows: 37 °C, 15 min and 85 °C, 5 s; the reaction was halt at 4 °C. The generated cDNA was stored in a −20 °C freezer. With cDNA as the template and U6 as the internal reference, RT-qPCR was performed with a real-time machine (7500 Real Time PCRSystem, ABI, Foster City, CA, USA) as well as a TaqMan MicroRNA Assay (4427975, Applied Biosystems, Foster City, CA, USA). The reaction conditions were comprised of pre-denaturation at 95 °C for 10 min, 30 cycles of denaturation at 95 °C for 10 s, annealing at 60 °C for 20 s and extension at 72 °C for 34 s, and staining with SYBRGreen fluorescent (RR091A, TaKaRa, Tokyo, Japan). The aforementioned method was also applicable to the cell experiment.

**Table 2:** Primer sequences of related genes for RT-qPCR.

Gene	Sequence
mmu-miR-495	Forward: 5′-AAACAAACATGGTGCA-3′
	Reverse: 5′-GAGCAGGCTGGAGAA-3′
AQP1	Forward: 5′-AGGCTTCAATTACCCACTGGA-3′
	Reverse: 5′-CTTTGGGCCAGAGTAGCGAT-3′
p38 MAPK	Forward: 5′-TGACCCTTATGACCAGTCCTTT-3′
	Reverse: 5′-GTCAGGCTCTTCCACTCATCTAT-3′
Cyclin D1	Forward: 5′-GCGTACCCTGACACCAATCTC-3′
	Reverse: 5′-ACTTGAAGTAAGATACGGAGGGC-3′
PCNA	Forward: 5′-TTGCACGTATATGCCGAGACC-3′
	Reverse: 5′-GGTGAACAGGCTCATTCATCTCT-3′
OCN	Forward: 5′-GGCTTAAAGACCGCCTACAG-3′
	Reverse: 5′-GAGAGGACAGGGAGGATCAA-3′
OPN	Forward: 5′-ATCTCACCATTCGGATGAGTCT-3′
	Reverse: 5′-TGTAGGGACGATTGGAGTGAAA-3′
RUNX2	Forward: 5′-GACTGTGGTTACCGTCATGGC-3′
	Reverse: 5′-ACTTGGTTTTTCATAACAGCGGA-3′
GAPDH	Forward: 5′-AGGTCGGTGTGAACGGATTTG-3′
	Reverse: 5′-GGGGTCGTTGATGGCAACA-3′
U6	Forward: 5′-CTCGCTTCGGCAGCACA-3′
	Reverse: 5′-AACGCTTCACGAATTTGCGT-3′

Note: RT-qPCR, reverse transcription quantitative polymerase chain reaction; miR-495, microRNA-495; p38 MAPK; p38 mitogen-activated protein kinase; PCNA, proliferating cell nuclear antigen; OCN, osteocalcin; OPN, osteopontin; RUNX2, runt-related transcription factor 2; GAPDH, glyceraldehyde-3-phosphate dehydrogenase.

Next, to determine the mRNA expression of each gene, cDNA was synthesized using High-Capacity cDNA Reverse Transcription Kit (4368814, Applied Biosystems, Foster City, CA, USA), with RT-qPCR conducted in accordance with the manuals of TaqMan Gene Expression Assays protocol (4331182, Applied Biosystems, Foster City, CA, USA). U6 was regarded as the internal reference. The reaction conditions consisted of pre-denaturation at 95 °C for 10 min, 30 cycles of denaturation at 95 °C for 15 s, annealing at 60 °C for 30 s and extension at 72 °C for 45 s. All RT-qPCR experiments were conducted with 3 duplicate wells, which was also applicable to the cell experiment.

### Western blot analysis

Tissues with liquid nitrogen were grinded in a uniform manner into fine powder, added with protein extraction kit (R0278, Sigma-Aldrich, St. Louis, MO, USA), centrifuged at 25764 × g for 20 min at 4 °C, after which the supernatant was collected for later use. The total protein concentration was estimated using bicinchoninic acid (BCA) protein assay kit (23250, Thermo Fisher Scientific, New York, USA) with the concentrations adjusted using deionized water in order to ensure an identical amount of samples. After the preparation of 10% sodium dodecyl sulfate (SDS) separation gel and concentrated gum, the sample was mixed with a sample buffer, boiled at 100 °C for 5 min, and subsequently transferred to the nitrocellulose membranes. The membranes were blocked with 5% skimmed milk powder overnight and then incubated at room temperature for 1 h with diluted rabbit monoclonal antibodies AQP1 (1: 1000, ab15080), p38 MAPK (1: 3000, ab197348), PCNA (1: 1000, ab92552), Cyclin D1 (1: 10000, ab134175), OCN (1: 500, ab93876), OPN (1: 1000, ab8448), and RUNX2 (1: 5000, ab92336) (Abcam, Cambridge, MA, USA). After 3 PBS washes (5 min per wash), the membranes were incubated with goat anti-rabbit IgG secondary antibody (1: 1000, ab6785, Abcam, Cambridge, MA, USA) at room temperature for 1 h, and then washed three more times with PBS (5 min per wash). An equal amount of A and B solutions were collected from the enhanced chemiluminescence (ECL) fluorescence detection kit (BB-3501, Amersham, Arlington Heights, IL, USA), mixed under conditions void of light and added onto the membranes. The images were captured in a MultiImager. Bio-Rad image analysis system (BIO-RAD, Foster, CA, USA) was used to photograph, and Image J software was employed for evaluation. Glyceraldehyde-3-phosphate dehydrogenase (GAPDH) was considered as the reference and the ratio of target protein band to internal reference band was regarded as the relative protein level.

### 3-(4, 5-dimethyl-2-thiazolyl)-2, 5-diphenyl-2-H-tetrazolium bromide (MTT) assay

The cells at the logarithmic growth phase were prepared into 2.5 × 10^5^ cells/mL suspensions using Roswell Park Memorial Institute (RPMI) 1640 medium containing 10% fetal bovine serum. The cells were seeded in a 96-well plate (100 μL per well) and cultured in a 5% CO_2_ incubator at 37 °C for 24 h, 48 h, and 72 h respectively. Following the addition of 10 μL MTT reagent (5 mg/mL) (Sigma-Aldrich, St. Louis, MO, USA) to each well, the cells were incubated for 4 h. With the MTT solution aspirated, 150 μL dimethyl sulfoxide (DMSO) was added into each well. Ten min later, OD at 490 nm was determined using a micro-plate reader (BIO-RAD, Foster, CA, USA) for each well. Each group had 3 parallel wells. The experiment was conducted three times to obtain the average value. A cell viability curve was drawn with time as the horizontal axis and the OD value as the vertical axis.

### Alizarin red staining

The cells in the logarithmic growth phase were counted and the cell concentration was then adjusted to 2 × 10^5^ cells/mL. The cells were then seeded into a 6-well plate for 21 days, washed three times with PBS, fixed by 40 g/mL paraformaldehyde for 10 min at room temperature, and carefully washed three times with double distilled water. After staining with 1% alizarin red S dyeing liquid for 5–10 min, the result was analyzed. The excess dye was subsequently discarded using a straw with the samples washed again for 3 times with double distilled water. Next, an inverted microscope was used to compare the staining results between the tibial fracture group and the control group, and five non-overlapping fields were randomly selected to photograph under a 400-fold microscope. The Alizarin red staining results predominately revealed red precipitation, with the cells displaying red precipitation regarded as calcium nodules.

### Alkaline phosphatase (ALP) activity assay

The cells from each group cultured for 7 days, 14 days and 21 days were detected by ALP with the operation performed on the ice. The cell culture medium in the 6-well plate was washed three times with PBS, after which the cells in each well were covered with 400 μL cell lysis buffer. The mixture was then fixed at 4 °C for 30 min to ensure the cells had been fully lysed. After lysis, the cells were scraped from one side to the other side and the lysate was taken into the pre-cooled EP tube and placed in the centrifuge tube containing ice. Next, ultrasonic wave was used to break the cell to ensure that the ultrasonic probe was below the level of the liquid (5 min per time, interval 5 s, five cycles). After 25764 × g centrifugation for 10 min at 4 °C, the supernatant was collected and placed into a new EP tube (not aspirated) and the ALP activity was detected at 520 nm wavelength. The experiment was repeated 3 times.

### Statistical analysis

Statistical analyses were conducted using SPSS 21.0 (IBM Corp., Armonk, NY, USA). Measurement data were expressed as mean ± standard deviation. Comparisons among multiple groups were conducted by one-way analysis of variance (ANOVA) and homogeneity test of variance. When the variance analysis had significant difference, the *q*-test was used for the comparison of two groups. Nonparametric rank test was applied when the variance was not uniform. The level of test taking alpha = 0.05, and *p* < 0.05 was considered to be reflective of statistical significance. Enumeration data were expressed as percentage or rate, while a chi-square test was used for comparison.
